# Oncological outcomes of rectal cancer patients with anastomotic leakage: A multicenter case-control study

**DOI:** 10.3389/fsurg.2022.993650

**Published:** 2022-09-12

**Authors:** Roberto Peltrini, Filippo Carannante, Gianluca Costa, Gianfranco Bianco, Giovanni Maria Garbarino, Giulia Canali, Paolo Mercantini, Umberto Bracale, Francesco Corcione, Marco Caricato, Gabriella Teresa Capolupo

**Affiliations:** ^1^ Department of Public Health, University of Naples Federico II, Naples, Italy; ^2^ Colorectal Surgery Unit, Fondazione Policlinico Campus Bio-Medico, Rome, Italy; ^3^ Department of Medical-Surgical Science and Translational Medicine, Sapienza University of Rome, Sant’Andrea Hospital, Rome, Italy

**Keywords:** rectal cancer, anastomotic leak, survival, recurrence, total mesorectal excision (TME)

## Abstract

**Introduction:**

This study aimed to evaluate the impact of anastomotic leakage (AL) on oncological outcomes after restorative rectal cancer surgery.

**Methods:**

Patients who underwent anterior resection for rectal adenocarcinoma between January 2011 and December 2017 were retrospectively reviewed. Data were collected from three colorectal surgery centers. Patients with grade B and C leaks according to the International Study Group of Rectal Cancer classification were identified and compared with the control group. Estimated recurrence and survival rates were compared using the log-rank method and Cox regression analysis.

**Results:**

A total of 367 patients were included in the study, with a mean follow-up of 59.21 months. AL occurred in 64 patients (17.4%). Fifteen patients with AL (23.5%) developed local recurrence (LR) compared to 17 (4.8%) in the control group (*p* < 0.001). However, distant recurrence rates were similar (10.9% vs. 9.6%; *p* = 0.914) between the groups. Kaplan-Meier curves showed that patients with AL had a reduced 5-years local recurrence-free survival (96% vs. 78%, log-rank *p* < 0.001). AL (OR 4.576; 95% CI, 2.046–10.237; *p* < 0.001) and node involvement (OR 2.911; 95% CI, 1.240–6.835; *p* = 0.014) were significantly associated with LR in multivariate analysis. AL was significantly associated with DFS only at univariate analysis (HR 1.654; 95% CI: 1.024–2.672; *p* = 0.037), with a difference between 5-year DFS of patients with and without AL (71.6% vs. 86.4%, log-rank *p* = 0.04). Only male gender, pT3-4 stage, and node involvement were identified as independent prognostic factors for reduced DFS in the multivariate Cox regression analysis.

**Conclusion:**

In this cohort of patients, AL was associated with a significant risk of LR after rectal cancer surgery.

## Introduction

Total mesorectal excision (TME), described by Heald (1, 2), decreases the local recurrence rate, underlying the importance of rectal dissection along embryological planes. Therefore, TME is currently considered the standard surgical treatment for mid–low rectal tumors. Likewise, preoperative chemoradiotherapy (CRT) improved oncological outcomes in locally advanced rectal cancers (3, 4).

However, radical surgery is associated with a high risk of perioperative complications, permanent stoma, and functional impairment (5). Although recent advances in rectal cancer management allow very low anastomosis promoting a sphincter-preserving strategy (6), anastomotic leakage (AL) rates range between 3% and 21% after rectal surgery (7, 8), with significant consequences on clinical and economic burden (9). Additionally, the impact of AL on oncological outcomes after anterior resection for rectal cancer remains controversial. Previous reports have identified no correlation between the incidence of AL and local recurrence or survival (10–12). However, some authors found impaired long-term oncological outcomes in patients with AL after anterior rectal resection (13, 14).

This study aimed to investigate the impact of AL on the recurrence and survival of patients undergoing sphincter-preserving surgery for rectal cancer.

## Materials and methods

We conducted a retrospective review to identify all patients who underwent restorative anterior rectal resection for adenocarcinoma at three different colorectal surgery centers from January 2011 to December 2017.

We included patients with histologically proven primary rectal tumors located within 15 cm of the anal verge who underwent surgery using an open or minimally invasive approach. Patients who did not meet the inclusion criteria, such as those treated in emergency settings or for palliative purposes, those who underwent rectal surgery for benign pathologies, those who had no restorative surgery (Hartmann's procedure or abdominal-perineal resection), those who underwent trans-anal TME or local excision with trans-anal endoscopic microsurgery (TEM) and patients lost to follow-up, were excluded.

The disease in all patients was staged using pelvic MRI, and chest and abdominal CT scans. Neoadjuvant chemoradiotherapy was performed for locally advanced mid–low rectal tumors (T3-4 and/or N+) followed by TME. For upper third rectal cancer, a mesorectal excision was performed by resecting from at least 5 cm below the distal margin of the tumor.

Baseline patient characteristics and cancer-related and operative data were collected from each participating center and successively merged in a comprehensive anonymized database.

Anastomotic leak was defined and classified according to the International Study Group of Rectal Cancer criteria (7). Grade A anastomotic leaks are identified by radiographic findings of perianastomotic fluid collection or leakage of contrast medium through the anastomosis without accompanying clinical complaints, and no active therapeutic intervention is required. Grade B leakage requires therapeutic interventions such as antibiotics and percutaneous drainage. Grade C anastomotic leakage requires reoperation. We considered only clinically relevant leaks (grades B and C) in the analysis. When postoperative clinical symptoms (fever, abdominal pain, ileus) and/or abnormal laboratory tests (leukocytosis, C-reactive protein) were observed, a CT scan assessment was performed to diagnose AL. All anastomotic dehiscence with leakage into the pelvic cavity and isolated pelvic abscesses with no evidence of fistula were considered ALs.

Oncological outcomes included disease-free survival (DFS), local recurrence (LR), and distant recurrence (DR), defined as the presence of a histopathologically proven or high radiological suspicion of tumor in the pelvis and outside the pelvis, respectively. Patients were followed-up every 3–6 months for the first 2 years after surgery and then every 6 months for a total of 5 years. CT scans of the thorax, abdomen, and pelvis; serum markers; and colonoscopy were performed according to the guidelines (15). Lost to follow-up is defined as a patient who has not received any contact with medical staff because of unavailability of updated patient data.

### Statistical analysis

Statistical analysis was performed using SPSS version 26 software (IBM Analytics Italia, Segrate, MI, USA) for Windows and StataCorp (2019) Stata Statistical Software Release 16 (College Station, TX: StataCorp LP). First, data normality was tested using the Shapiro-Wilk or Kolmogorov-Smirnov tests. Data and counts for dichotomous variables were presented as frequencies. Continuous data were presented as mean ± one standard deviation (SD) or as median and interquartile (25%–75%) or minimum–maximum range. To compare differences in frequencies, Fisher's exact test or the *χ*2 test with or without Yates correction was performed. Differences between means were compared using the Mann-Whitney U test and Student’s t-test. Univariate and multivariate forward stepwise logistic regression model (minimum AIC) were performed considering local recurrence as binary dependent variable. Survival time data of local recurrence and DFS were estimated using the Kaplan-Meier method and differences were analyzed using the log-rank Mantel-Cox test. Multivariate analysis of DFS was then performed using Cox logistic regression model. Only variables with *p* value <0.2 at univariate analysis were entered in multivariate models. The results were reported as Odds Ratio (OR) or Hazard Ratio (HR) with 95% confidence intervals, when appropriated. Statistical significance was set at *p* < 0.05.

## Results

In total, 419 patients underwent restorative rectal cancer surgery between January 2011 and December 2017. The study population included 367 patients because 52 patients (12.4%) were lost to follow up.

The baseline patient characteristics and perioperative details are described in Table 1. Tumors were located in the mid and low rectum in 63.2% of cases. AL occurred in 64 patients (17.4%). In each center AL was 17.8%, 16.2% and 17.6%, respectively. Patients were equally distributed between the groups for mean age, BMI, ASA score, tumor location, and comorbidity rate. Similarly, no differences in nCRT and surgical parameters were recorded. However, there was a significantly higher proportion of male patients in the AL group (*p* = 0.007), and the pathological T stage was more advanced (Table 2).

**Table 1:** Baseline patients’ characteristics and perioperative features.

	Total *n* = 367 (%)	Leak Group *n* = 64 (%)	No Leak Group *n* = 303 (%)	*p*
Age y mean (± SD)	68.5 (±11.5)	65.17 (±13.25)	68.50 (±10.25)	0.84
Gender				
Male	218 (59.4)	48 (75)	170 (56.1)	**0.007**
Female	149 (40.6)	16 (25)	133 (43.9)	
ASA score				
1/2	199 (54.2)	32 (50)	167 (55.1)	0.49
3/4	168 (45.8)	32 (50)	136 (44.9)	
BMI (Kg/m^2^) Mean (± SD)	25.4 (±4.08)	25.81 (±4.19)	25.16 (±2.99)	0.52
Comorbidity	201 (54.7)	37 (57.8%)	164 (54.1)	0.58
Tumor distance from a.v.				
>10 cm	135 (36.8)	20 (31.25)	115 (38)	0.56
5.1–10 cm	164 (44.7)	32 (50)	132 (43.5)	
<5 cm	68 (18.5)	12 (18.75)	56 (18.5)	
nCRT	165 (44.9)	30 (46.9)	135 (44.5)	0.88
Surgical approach				
Open	122 (33.2)	22 (34.4)	100 (33)	0.81
Laparoscopic	241 (65.6)	41 (64)	200 (66)	
Robotic	4 (1.2)	1 (1.6)	3 (1)	
Diverting ileostomy	183 (49.8)	42 (46.9)	141 (47)	0.43

nCRT, neoadjuvant chemoradiotherapy.

Bold values indicate statistical significance.

**Table 2:** Pathological details.

	Total *n* = 367 (%)	Leak Group *n* = 64 (%)	No Leak Group *n* = 303 (%)	*p*
pT				
pT0	43 (11.9)	6 (9.4)	37 (12.5)	**0.04**
pT1	38 (10.5)	9 (14)	29 (9.6)	
pT2	71 (19.4)	5 (7.8)	66 (21.8)	
pT3	191 (51.5)	37 (57.8)	154 (50.8)	
pT4	24 (6.7)	7 (11)	17 (5.3)	
pN				
pN0	240 (65.4)	38 (59.4)	201 (66.5)	0.26
pN1	84 (22.9)	16 (25)	68 (22.5)	
pN2	43 (11.7)	10 (15.6)	34 (11)	
Harvested lymph nodes mean (± SD)	20.52 (±10.6)	22 (±9.6)	20.3 (±10.6)	0.33
Stage				
I-II	240 (65.4)	38 (59.4)	202 (66.7)	0.27
III-IV	127 (34.6)	26 (40.6)	101 (33.4)	

Bold values indicate statistical significance.

The oncological outcomes are shown in Table 3. Fifteen patients with AL (23.4%) developed LR compared with 17 (4.8%) in the control group (*p* < 0.001). However, DR rates were similar between the groups (10.9% vs. 9.6%; *p* = 0.914).

**Table 3:** Oncological outcomes.

	Total *n* = 367 (%)	Leak Group *n* = 64 (%)	No Leak Group *n* = 303 (%)	*p*
Distant recurrence	41 (11.1)	7 (10.9)	34 (9.6)	0.914
Local recurrence	32 (8.7)	15 (23.4)	17 (4.8)	**<0.001**
Follow-up (months)				
mean (± SD)	59.21 (±25.09)	58.13 (±26.78)	59.40 (±24.81)	0.708
median (IQR)	58.65 (37.87–77.22)	60.18 (37.10–77.18)	58.12 (38.72–77.22)	

Bold values indicate statistical significance.

Only AL (OR 4.576; 95% CI, 2.046–10.237; *p* < 0.001) and node involvement (OR 2.911; 95% CI, 1.240–6.835; *p* = 0.014) were significantly associated with LR in multivariate analysis. (Table 4).

**Table 4:** Univariate and Multivariate analysis of Local Recurrence.

	Univariate Analysis		Multivariate Analysis	
* *	*OR* (*95% CI)*	*p*	*OR* (*95% CI)*	*p*
Age (>65y)	*1.014* (*0.981*–*1.048)*	*0*.*407*	* *	* *
Gender				
Female	* *	* *	* *	* *
Male	2.127 (0.923–4.898)	0.069		
Anastomotic leak				
No				
Yes	5.150 (2.357–11.250)	**<0.001**	4.576 (2.046–10.237)	**<0.001**
pT				
T0. Tis. T1. T2				
T3. T4	1.726 (0.768–3.876)	0.180		
pN				
N0		** **		
N+	2.986 (1.375–6.487)	**0.003**	2.911 (1.240–6.835)	**0.014**
nCRT				
No				
Yes	1.644 (0.789–3.425)	0.18		

OR, odd ratio.

Bold values indicate statistical significance.

**Figure 1:**
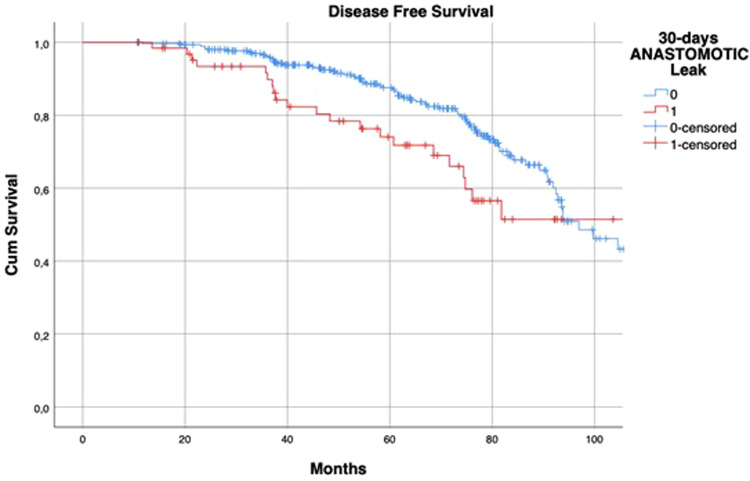
Kaplan–Meier curves of local recurrence-free survival, according to the occurrence of postoperative anastomotic leakage. *p* < 0.001 (log rank test).

The median follow-up was 60.18 (37.10–77.18) months in the AL group and 58.12 (38.72–77.22) months in the control group (*p* = 0.708). Kaplan-Meier curves showed that patients with AL had a reduced 5-year LRFS (96% vs. 78%, log-rank *p* < 0.001) (Figure 1).

**Figure 2:**
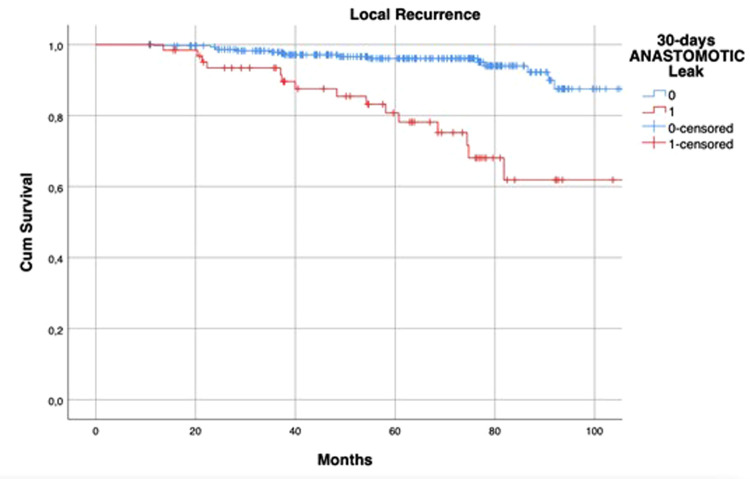
Kaplan–Meier curves of disease-free survival, according to the occurrence of postoperative anastomotic leakage. *p* = 0.04 (log rank test).

HR of AL regarding DFS at univariate analysis was statistically significant (HR: 1.654; 95% CI: 1.024–2.672; *p* = 0.037) but this was not confirmed at multivariate analysis (Table 5). The 5-year DFS of patients with leakage was different (71.6% vs. 86.4%, log-rank *p* = 0.04) when compared to that of the control group (Figure 2).

**Table 5:** Univariate and Multivariate analysis of Disease-free survival.

	Univariate Analysis	*p*	Multivariate Analysis	*p*
	HR (95% CI)		HR (95% CI)	
Age (≤65y; >65y)	1.009 (0.988−1.030)	0.393		
Gender				
Female				
Male	1.821 (1.097−3.024)	**0.019**	1.947 (1.938−3.331)	**0.015**
Anastomotic leak				
No				
Yes	1.654 (1.024−2.672)	0.037		
pT				
T0. Tis. T1. T2				
T3. T4	2.760 (1.597−4.768)	**<0.001**	2.078 (1.152−3.747)	**0.015**
pN				
N0				
N+	3.706 (2.212−6.487)	**<0.001**	2.992 (1.767−5.067)	**<0.001**
nCRT				
No				
Yes	1.240 (0.774−1.986)	0.396		

HR, hazard ratio.

Bold values indicate statistical significance.

Only male gender, pT3-4 stage, and node involvement were identified as independent prognostic factors for reduced DFS in the multivariate Cox regression analysis (Table 5).

## Discussion

This study aimed to evaluate the relationship between AL and oncological outcomes in patients with rectal cancer who underwent restorative surgery. We found that AL significantly affected the LR rate and LRFS, whereas it had no impact on DR. Although a difference in DFS was detected between the groups, multivariate analysis revealed that DFS was not affected by AL.

Previous studies have reported contradictory results regarding this issue. Data from the Memorial Sloan-Kettering Cancer Center, including 1,127 rectal cancer patients covering a period of almost 20 years, showed that the presence of AL did not change the risk of LR and disease specific or overall survival (10). The authors also clarified that this finding was independent of the presence of a defunctioning stoma. Likewise, a single-center study of 698 patients demonstrated that AL was not a significant independent risk factor for recurrence and survival in patients who underwent preoperative chemoradiotherapy (12). Even in a multicenter observational study using data from 1,181 patients from the Spanish Rectal Cancer Project database, the relationship between AL and long-term oncologic outcomes was mitigated (16). Furthermore, the retrospective analysis by Crippa et al. (17) from Mayo Clinic did not find any negative prognostic impact of AL (a standardized definition of AL was used) in a cohort of 787 patients, and both LR and symptomatic AL rates were very low (2% and 5.3%, respectively).

Despite the evidence suggested by the reports of these influential institutions, our results are consistent with those of a recent meta-analysis involving 11,353 patients (13). Only studies that analyzed the impact of AL on long-term outcomes using a multivariate Cox proportional hazards model were included. The authors reported a greater local recurrence rate (HR: 1.71; 95% CI: 1.22–2.38; *p* = 0.002) and decreased cancer-specific survival (HR: 1.30; 95% CI: 1.08–1.56; *p* = 0.005) in patients with AL. Additionally, AL did not increase DR (HR: 1.03; 95% CI: 0.76–1.40; *p* = 0.86), as we demonstrated in the present study. The association of AL with LR after rectal resection was also confirmed in the most recent similar systematic reviews (18, 19) and in other relevant single or multi-institutional reports (14, 20). Finally, our findings are consistent with long-term data analysis of the COLOR II trial (21), where an increase in LR (13.3% vs. 4.6%; HR: 2.96; 95% CI: 1.38–6.34; *p* = 0.005) and a decrease in DFS (53.6% vs. 70.9%; HR: 1.67; 95% CI: 1.16–2.41; *p* = 0.006) at the 5-year follow-up were found in patients with AL. Similar to our results, AL was not a significant predictor of DR (HR: 1.21, 95% CI: 0.71–2.04).

The mechanism by which AL increases LR after rectal cancer surgery remains unclear. Postoperative sepsis may induce an inflammatory response. Some data suggest that the systemic inflammatory response participates in the progression of metastatic disease in patients with colorectal cancer (22). The release of proinflammatory cytokines and growth factors as part of the systemic inflammatory response secondary to intra-abdominal sepsis and the associated immunosuppression have direct effects on the growth of residual tumor cells (23). In fact, IL-1beta and TNF-alpha are significant stimulating factors in tumor cell adhesion *in vitro* and may therefore affect tumor recurrence to the peritoneum *in vivo* (24). Furthermore, it has been demonstrated that postoperative sepsis could lead to a period of immunosuppression, resulting in proliferation of the metastatic tumor cells (25). Therefore, the immunosuppressive status induced by septic complications and AL may lead to unfavorable oncological outcomes (26, 27).

Otherwise, AL might lead to local implantation of viable cancer cells at the anastomotic site at the time of surgery (28, 29). Finally, a delay in initiating adjuvant treatment due to prolonged length of hospital stay can affect survival in patients with colorectal cancer (30).

On the other hand, risk factors for AL such as male gender, obesity, previous radiotherapy and T stage are well established (8, 31). We detected a significantly greater proportion of male patients and more advanced tumors in the AL group. In contrast, there was no difference in diverting ileostomy construction between the groups. This may support the hypothesis that a defunctioning stoma decreases the clinical severity of AL rather than prevents anastomotic complications (8, 10, 32).

Although we found an AL rate of 17.4%, which is slightly higher than the 9–11% published elsewhere (16, 33, 34), the value is consistent with the current literature reporting a prevalence of AL between 3% and 21% after restorative anterior resection (7, 31). Furthermore, the LR rate in the present study was 8.7% (32/367), similar to that in the French single institutional series of 428 patients (8.4%) (14) and that of the Swedish Rectal Cancer Registry with 250 patients (8%) (11). This contributes to the external validity of our study.

This study has some limitations. The data were retrospectively collected, which has the risk of patient selection bias. We only included patients with grade B and C leaks because routine postoperative imaging was not performed to detect asymptomatic leaks. Furthermore, the diverting stoma was performed according to the surgeon's preference and no details regarding adjuvant chemotherapy or other phatological features were provided.

## Conclusion

Anastomotic leakage contributes to adverse oncologic outcomes such as LR after restorative rectal cancer surgery. Therefore, prevention to minimize the risk is essential, and careful surveillance and tailored oncologic assessments should be considered.

## Data Availability

The original contributions presented in the study are included in the article/Supplementary Material, further inquiries can be directed to the corresponding author/s.
